# Identifying SNARE Proteins Using an Alignment-Free Method Based on Multiscan Convolutional Neural Network and PSSM Profiles

**DOI:** 10.1021/acs.jcim.2c01034

**Published:** 2022-09-27

**Authors:** Quang-Hien Kha, Quang-Thai Ho, Nguyen Quoc Khanh Le

**Affiliations:** † International Master/Ph.D. Program in Medicine, College of Medicine, Taipei Medical University, Taipei 110, Taiwan; ‡ College of Information & Communication Technology, Can Tho University, Can Tho 90000, Viet Nam; § Department of Computer Science and Engineering, Yuan Ze University, Chung-Li 32003, Taiwan; ∥ Professional Master Program in Artificial Intelligence in Medicine, College of Medicine, Taipei Medical University, Taipei 106, Taiwan; ⊥ Research Center for Artificial Intelligence in Medicine, Taipei Medical University, Taipei 106, Taiwan; # Translational Imaging Research Center, Taipei Medical University Hospital, Taipei 110, Taiwan

## Abstract

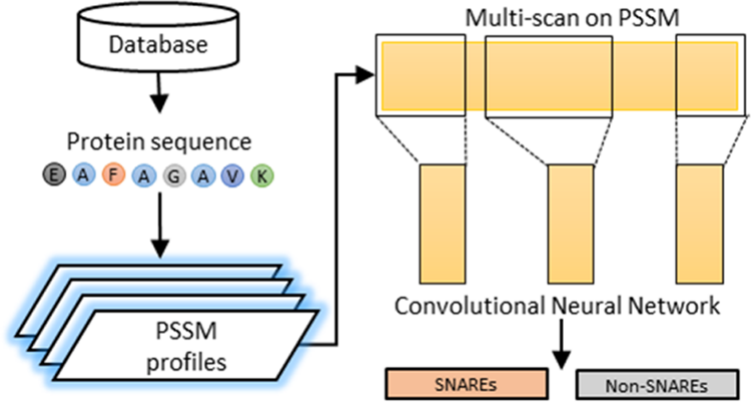

*Background*: SNARE proteins play a vital role in membrane fusion and cellular physiology and pathological processes. Many potential therapeutics for mental diseases or even cancer based on SNAREs are also developed. Therefore, there is a dire need to predict the SNAREs for further manipulation of these essential proteins, which demands new and efficient approaches. *Methods*: Some computational frameworks were proposed to tackle the hurdles of biological methods, which take plenty of time and budget to conduct the identification of SNAREs. However, the performances of existing frameworks were insufficiently satisfied, as they failed to retain the SNARE sequence order and capture the mass hidden features from SNAREs. This paper proposed a novel model constructed on the multiscan convolutional neural network (CNN) and position-specific scoring matrix (PSSM) profiles to address these limitations. We employed and trained our model on the benchmark dataset with fivefold cross-validation and two different independent datasets. *Results*: Overall, the multiscan CNN was cross-validated on the training set and excelled in the SNARE classification reaching 0.963 in AUC and 0.955 in AUPRC. On top of that, with the sensitivity, specificity, accuracy, and MCC of 0.842, 0.968, 0.955, and 0.767, respectively, our proposed framework outperformed previous models in the SNARE recognition task. *Conclusions:* It is truly believed that our model can contribute to the discrimination of SNARE proteins and general proteins.

## Introduction

First identified in 1980, SNARE (soluble *N*-ethylmaleimide-sensitive factor attachment protein receptor) proteins specify a superfamily group of small proteins containing a characteristic structure of SNARE-motif with 60–70 amino acids arranged in heptad repeat order.^1^ In eukaryotes, SNAREs aid in the catalyzation of membrane fusion and mediate in various cellular living processes such as cell proliferation, cell division, and neurotransmission.^1,2^ Based on the cellular locations and functionalities, SNARE proteins are divided into two groups including v-SNAREs (vesicle membrane) and t-SNAREs (target membrane).^3,4^ The VAMPs (synaptic vesicle-associated membrane proteins or synaptobrevin) reside on the synaptic vesicle,^5^ while syntaxin-1 and synaptosomal-associated protein 25 kDa (SNAP-25) are presynaptic membrane proteins.^6−8^ Both VAMP and syntaxin have their C-terminal residues inserted in the membrane, whereas the palmitoylated cystein residues in the central zone helps SNAP-25 bind to the plasma membrane.^5,9,10^ By far, many SNARE proteins have been discovered and the presence, absence, or impairment of SNAREs involved in the pathological process or even potential therapeutics of cancer,^11−13^ neurodegenerative diseases,^14,15^ psychiatric disorders,^16,17^ and more. With the importance of SNAREs in the functionality of cells and the body, finding new approaches that can robustly identify, classify, and predict their functions is a necessity.

A plethora of recent biological studies have been conducted to predict the functions of different SNARE proteins. Gao et al.^18^ explored the role of SNARE Ykt6 in membrane fusion during autophagy in yeast cells^19^ and demonstrated the importance of SNARE Sec. 22b in embryonic development, as lacking this protein can lead to uterus death in experimented mice. SNAP-25 mutants may inhibit the synaptic membrane fusion in botulinum infection pathology.^20^ Despite the significant findings, these studies take much time and budget to complete the procedure, also the framework remains hard to replicate in real-world practice. With the development of machine learning algorithms, different kinds of proteins and their functions can now be identified and predicted using the computational methods.^21^ For SNARE proteins, Le and Nguyen,^22^ as pioneers in this field, have ensembled a model and web server termed SNARE-CNN based on convolutional neural network (CNN), with a newly proposed benchmark dataset of SNARE sequences. To date, various studies have been conducted on the aforementioned dataset to improve the predictive performance using different methods such as Manhattan distance and k-nearest neighbors (kNN),^23^ hybrid model,^24^ or support vector machine–recursive feature elimination–correlation bias reduction (SVM-RFE-CBR).^25^

However, all current methods approaching the prediction problem face two independent issues. First, most previous studies used conventional machine learning (ML) algorithms, which could not retrieve the hidden information from sequence information compared to deep learning (i.e., CNN). Motivated by the human brain,^26^ CNN assembled and unbroken the limitations of traditional ML techniques to become a robust tool for image classification,^27^ protein prediction,^28,29^ and so on. Various studies have indicated the capabilities of CNN in extracting the underlying features deep within the input data, from which we can perform the prediction or identification of components more effectively.^30,31^

Another limitation of previous studies identifying SNARE proteins is that, if the study exploited CNN, they could not keep the sequencing order in position-specific scoring matrix (PSSM) profiles in the model, which was previously observed in SNARE-CNN study.^22^ To avoid the loss of position and order information in the protein sequences, Ho et al.^31^ has proposed a novel approach utilizing the feedforward CNN^32^ with a multiple window scanning technique. They also used the whole PSSM profiles as input data to assure that the position and order of the amino acids in the sequences would be kept stable during the training process. This leads to broader generalizability of the protein sequences, and based on this, the model may give a more precise prediction compared to conventional CNN frameworks.

Given the above considerations, we herein propose a novel deep learning framework based on multiscan CNN and PSSM profiles of the SNARE proteins to address the hurdles of the previous SNARE classifiers and improve the prediction performance on SNARE proteins. In detail, we transformed the FASTA-formatted SNAREs into PSSM profiles, which were then fed into the 20-channel networks (i.e., corresponding to 20 amino acids). We architected the layers of the multiscan CNN, combining different window sizes to extract the most features out of each profile. We prepared one cross-validation set and two independent test sets to measure our model′s efficiency meticulously. Furthermore, a precise comparison between our proposed architecture and other existing methods was made to demonstrate the supremacy in the SNARE prediction task yielded by our model.

## Materials and Methods

**Figure 1:**
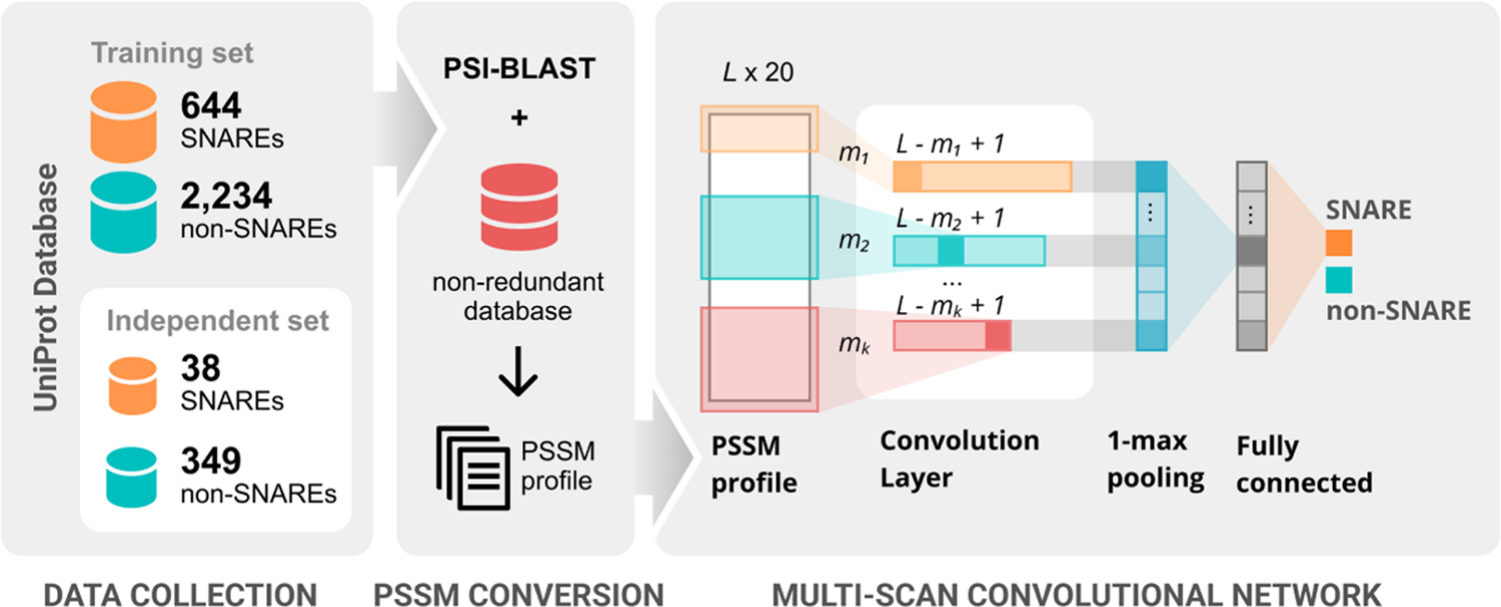
Flowchart of our proposed method.

Figure 1 illustrates our proposed method including different subprocesses: data collection, feature engineering, model implementation, and performance evaluation. In detail, we first prepared one cross-validation dataset (i.e., for training the model) and one independent test set. We next constructed the PSSM profiles of all SNARE sequences and formulated the design of the multiscan CNN framework. Finally, we certified the identification performance of our model on SNARE proteins with experimental metrics, visualization methods and graphs, and comparative tables versus other models.

### Benchmark Dataset

To ensemble a model that can precisely recognize the SNARE proteins, it is of importance to have an appropriate dataset. We referenced the benchmark dataset presented by Le and Nguyen,^22^ which contains 682 SNARE proteins and 2583 non-SNARE proteins. In detail, this benchmark study looked for the protein sequences with keyword “SNARE” from the UniProt database,^33^ which contains extensive and comprehensive information about protein sequences. They later applied the BLAST^34^ to remove all redundant sequences, and sequences with similarity over 30% appeared in the results. Eventually, 682 SNARE sequences were included in the training set as the positive samples. For the negative representatives, we followed the procedure in Le and Nguyen^22^ and retrieved 2583 general proteins that were not SNAREs. The previous study also split the primary dataset into a cross-validation set (i.e., 644 SNAREs and 2234 non-SNAREs) and an independent set #1 (i.e., 38 SNAREs and 349 non-SNAREs) to implement further experiments.

Moreover, we used the same strategy to manually collect another dataset from UniProt^33^ which contained newly discovered proteins (discovered from November 1, 2018 to August 1, 2022). This idea aimed to get SNAREs and non-SNAREs that have not yet appeared in the paper′s benchmark data. This dataset, namely, independent dataset #2, contained 15 SNAREs and 126 non-SNAREs and is used as an external validation dataset to evaluate the performance of model. Table 1 shows detailed statistics of our full dataset.

**Table 1:** Detailed Statistics of Dataset Used in this Study^a^

	training data	independent data #1	independent data #2
SNAREs	644	38	15
non-SNAREs	2234	349	126

a Training data and independent data #1 were retrieved from the previous study.^22^ Independent data #2 is newly discovered data (from November 1, 2018) that were manually collected in this study.

### Feature Engineering

#### PSSM Profiles

As aforementioned, it is important to architect the model on a proper feature extraction method to distinguish the SNARE sequences among vesicular transporting proteins. We applied the PSSM profile, which was proposed by Jones^35^ and successfully employed in various bioinformatics research (e.g., protein function prediction,^36,37^ subcellular localization prediction,^38^ protein secondary structure prediction,^39^ and so on), to extract the underlying features of SNARE proteins later used as the CNN’s training attributes.

Each PSSM profile was made up of a matrix with *L* rows and *N* columns, with *L* equal to the input sequence length and *N* for 20 amino acids. First, we conditionally summed up the rows which belonged to the same amino acid to generate a 20 × 20 matrix, i.e., a new (20 × 20)-dimensional PSSM profile. Each element in the (20 × 20) matrix was next divided by the window size *W* and normalized by the sigmoid function before feeding into the multiscan CNN
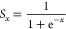
1

We imposed conversing all FASTA-formatted SNARE proteins in the original data to PSSM profiles by utilizing PSI-BLAST^34^ to filter out the FASTA sequences in the nonredundant (NR) database^40^ with three iterations and accomplish the conversion.

#### Other Sequence-Based Features

A plethora of feature extraction methods were conducted to generate many models to identify different types of proteins.^41,42^ We also employed well-known sequence-based features in bioinformatics to compare their performance with raw PSSM profiles.

Amino acid composition (AAC) is used to convert a protein sequence into an array of 20 elements containing the frequencies of amino acid residues in the input sequence.

Pseudo amino acid composition (PAAC) is an improvement to the shortcoming of sequence loss resulting from the conventional AAC by adding the information about sequence order via pseudo components.

Dipeptide composition (DPC) converts the protein sequence to a 2D array by (20 × 20) containing the frequency of occurrence of each amino acid pair in the sequence.

Amphiphilic pseudo amino acid composition (APAAC) has the same form as the conventional AAC, but it provides more information regarding the sequence order of one protein including where the hydrophobic and hydrophilic amino acids cross the chain.

Grouped amino acid composition (GAAC) calculates the frequency of each amino acid group. The 20 different amino acid residues are clustered into five groups (i.e., five dimensions) using their physicochemical properties.

Composition of k-spaced amino acid pairs (CKSAAPs) reflects the short-range interactions of amino acids within a sequence or sequence fragment.

Composition of k-spaced amino acid group pairs (CKSAAGPs) reflects the short-range interactions of residues within a sequence or a sequence fragment.

### Model Architecture

In this section, we focused on describing the structure of our proposed method, which aimed for the robust recognition of SNARE proteins. Based on the principle of multitask learning and following the architecture of DeepFam,^32^ the design of our model architecture was constructed on multiscan CNN including various convolutional layers. Inspired by the performance of DeepFam, this multiscan CNN has been also applied in the later sequence-based studies such as electron transporters^31^ or ion transporters.^43^

The layers were designed with different window sizes *L* of (16, 24, 32) to recognize the patterns better for the prediction task. We input the sequences of the (20 × 20) matrix into the convolution layer, which scanned those sequences across 20 channels. The operation continued by windowing each convolution unit over the sequences. Each transformed sequence with length *L* was output at the convolution layer of which the size was reduced to *L* – *W*_k_ + 1 (*W*_K_ is the size of each convolution unit). We recruited the ReLU (rectified linear unit) activation function for all hidden layers, which was formulated as
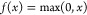
2

For each filter output, we attempted to keep only the most superior attention. Thus, we employed 1-max pooling layer^44^ at the end of each convolution layer with the formula of
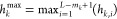
3

### Performance Evaluation

The model was fivefold cross-validated on the training set, i.e., first splitting the dataset into five subsets, and one of them would be used as the testing set while others were for training purpose, respectively, to evaluate its performance on the SNARE recognition task. Thereafter, the model was evaluated on two different independent datasets. Statistically, we validated the robustness of the SNARE detection performance based on several metrics, i.e., accuracy (ACC), sensitivity (Sens), specificity (Spec), and Matthews correlation coefficient (MCC)
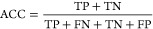
4
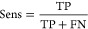
5
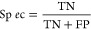
6
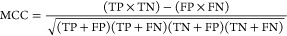
7

where TP, FN, TN, and FP denotes true positive, false negative, true negative, and false negative, respectively. We also would like to verify the competency of our model and compare it with other frameworks in discriminating the SNARE and non-SNARE sequences; thus, we plotted the receiver operating characteristic (ROC) curve and precision–recall (PR) curve.

## Results and Discussion

### Model Selection and Parameter Optimization

During the training process, our model was trained and cross-validated to observe its initial efficiency. Because our datasets were imbalance, synthetic minority oversampling technique (SMOTE)^45^ was applied aiming to achieve a better performance in sensitivity. It is noticed that we only applied oversampling on training data and kept original data in testing data as well as two independent datasets. It is necessary to advance model′s performance through processing the hyperparameter tuning. The fivefold cross-validation continued with different combinations of parameters as we took into account some parameters for tuning, i.e., epoch of (10, 50, 100), batch size of (10, 50, 100), and learning rate of (0.0001, 0.0001, 0.001). The area under the ROC curve (AUC) score was recorded and used to determine which set of parameters would be chosen to generate the optimal model. After the experiment, the best performance of the multiscan CNN could be achieved at the epoch of 10, batch size of 10, and learning rate of 0.0001.

### Baseline Comparison

It is important to demonstrate the superiority of our framework over the existing computational methods in identifying the SNARE proteins. Therefore, we employed other renowned feature extractors and classifiers for the performance comparison. For the former, we used the same CNN architecture to learn different features to see the performance among them. It can be observed in Table 2 that our PSSM features outperformed other features in most measurement metrics. In detail, we could achieve a sensitivity of 84.5%, specificity of 95.5%, accuracy of 93.0%, and MCC of 0.800 in the cross-validation experiments. The conventional AAC and DPC features gave the highest sensitivity scores, which showed that these features excelled in detecting the true SNARE proteins. Various studies in literature have utilized Chou’s AAC^41,46^ and DPC^47,48^ to predict different types of proteins explaining why these feature extractors worked well on SNAREs prediction. Nonetheless, their overall MCC metrics were noticeably lower than that of our method. Despite the slightly low sensitivity, our method using a PSSM feature extractor could hit the highest MCC value (0.800), which indicates the high predictive efficiency for this imbalanced benchmark dataset and binary problem (i.e., distinguishing between SNARE and non-SNARE proteins).^49,50^

**Table 2:** Comparison to Other Sequence-Based Features in Protein^a^

feature	sensitivity	specificity	accuracy	MCC
AAC	0.844	0.811	0.814	0.430
DPC	0.838	0.860	0.858	0.492
PAAC	0.782	0.877	0.869	0.485
APAAC	0.781	0.883	0.874	0.493
GAAC	0.755	0.718	0.721	0.285
CKSAAP	0.824	0.872	0.868	0.502
CKSAAGP	0.802	0.805	0.805	0.397
PSSM	0.845	0.955	0.930	0.800

a All of the results were obtained using CNN architecture on the training set via a cross-validation scheme. SMOTE algorithm was applied to resolve imbalance problems.

**Figure 2:**
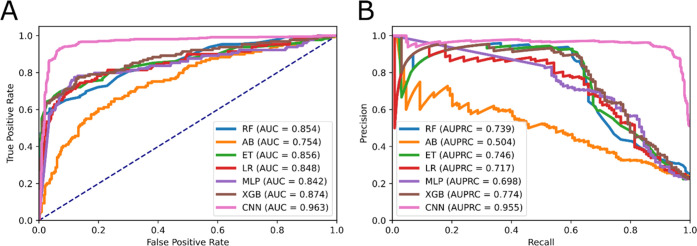
Comparison among different models. (A) ROC curve and (B) precision–recall Curve.

Six classifier algorithms, i.e., Random Forest (RF), Adaptive Boosting classifier (AB), Extra Tree Classifier (ET), Logistic regression (LR), Multilayer perceptron classifier (MLP), and eXtreme Gradient Boosting (XGB), were selected to make the performance comparison with our proposed model. We trained and tested the classifiers on the same training set that we applied multiscan CNN. Fivefold cross-validation was undergone to make sure that the results were reliable and comparative. As can be observed from Figure 2, the CNN surpassed other classifiers, as its performance attained the top AUC and AUPRC of 0.963 and 0.955, respectively. With these promising results, we strongly believe that we are capable of constructing an optimal architecture for this kind of feature data.

### Independent Tests

To see the potential of overfitting or overoptimistic performance, we inserted two different independent datasets into our trained model to see their performance. The results then showed a sensitivity of 0.842, specificity of 0.968, accuracy of 0.955, and MCC of 0.767 in the independent dataset #1. For the independent dataset #2, our model achieved a sensitivity of 0.8, specificity of 0.952, accuracy of 0.936, and MCC of 0.7. Compared to the cross-validation results (in Table 2), they reached a very similar performance, and it convinces that the model did not rely on overfitting problem.

### Visualization of Deep PSSM Features

**Figure 3:**
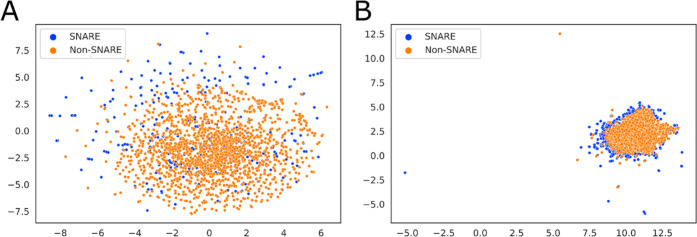
Feature representation of multiscan PSSM profiles. (A) t-SNE analysis and (B) UMAP analysis.

To better interpret the model performance made by neural networks, we use uniform manifold approximation and projection (UMAP) and t-distributed stochastic neighbor embedding (t-SNE) to visualize the hidden features. t-SNE^51^ and UMAP^52^ are used to reduce the dimensions of input data, and they both aid in better understandings about underlying features of high-dimensional data by visualizing these types of data into two-dimensional maps, thereby significantly deducting the perplexity of data. As shown in Figure 3, we extracted the final classification representations (the output of final layers) and depicted them in two dimensions. In Figure 3A, the SNAREs and non-SNAREs were well classified by the model construction of multiscan CNN and PSSM profiles. However, the blue and orange points, which symbolized the sequences in the input space, were not well separated in t-SNE analysis, resulting in unclear depiction. Thus, there is a need to perform another visualization method to enhance the interpretation. We subsequently performed UMAP analysis, and the distinguishment between two classes of protein sequences was explicitly portrayed in Figure 3B. The features displayed by t-SNE and UMAP proved the prediction power of our proposed framework in discriminating SNARE sequences among general proteins.

### Comparison to Previously Published Works

Since the publication of the benchmark dataset,^22^ the identification of SNARE proteins has gained much interest from researchers. In addition to the deep learning framework by Le and Nguyen,^22^ current method proposed by SNAREs-SAP^25^ architected on machine learning algorithms also achieved high performances on SNAREs data. In this section, we focused on comparing the predictive efficiency made by our model with aforementioned ones since we used the same dataset. As we can notice from Table 3, our method outperformed other models in most metrics. In detail, our specificity and accuracy reached the top of 0.974 and 0.946, respectively.

**Table 3:** Comparison to Previous Predictors Using the Same Independent Dataset^a^

predictor	sensitivity	specificity	accuracy	MCC
SNARE-CNN^22^	0.658	0.903	0.879	0.460
SNAREs-SAP^25^	0.680	0.940	0.920	0.480
proposed method	0.842	0.968	0.955	0.767

a All of the results were obtained on independent dataset #1.

In the original study, Le and Nguyen^22^ employed CNN to train their model and PSSM profiles to extract the interested features, which were similar to our method. However, one drawback was that their two-dimensional CNN (2D-CNN) architecture could not maintain the order of input sequences. Unlike 2D-CNN, multiscan CNN was competent in retaining the sequence at their basic order facilitating the learning process of the algorithms and broaden the probability of correct prediction. As a result, the MCC obtained from our model increased more than 1.67-fold from 0.460 yielded by SNARE-CNN.

SNAREs-SAP, which was developed by Zhang et al.,^25^ assembles from SVM-RFE-CBR and PSSM profiles. Similarly, the architecture of CKSAAP-Manhattan^23^ was constructed on a kNN classifier, and its feature extraction was based on the CKSAAP method. Both SVM and kNN are two of the most common methods in bioinformatics; they have been applied widely as baseline algorithms in frameworks that perform excellently in terms of subcellular organism detection,^38,47^ protein functional prediction,^53,54^ and so on. However, with the capabilities of unsupervised learning from high-throughput and multidimensional data, deep learning has been evidenced to surpass traditional machine learning algorithms in performing robust protein function prediction.^37,55^ This is owing to the ability of extracting hidden features,^56,57^ thereby gaining comprehensive estimation and clustering the input sequences based on original and additional features.

On top of that, for interdisciplinary research field like bioinformatics, where large datasets are intriguingly available and getting easier to access, the implication of deep learning is believed to be more suitable compared to conventional machine learning methods.^30^ This is also true in the task of SNARE recognition, with a large size of high-dimensional data, where our framework achieved reasonably high experimental metrics using CNN.

In bioinformatics research, not only the selection of baseline algorithm is important but how we extract the data features also matters. So far, there has not been a true comparison between the efficiency of PSSM profiles and other feature extraction techniques. However, in this study, we experimented constructing not only the PSSM-based model but also using the renowned techniques, including CKSAAP. The measurements in Table 2 indicated that the PSSM profiles can assist better predictive performance on SNAREs than features extracted by CKSAAP techniques. Taken together, our model architecture approached a deep learning strategy with feedforward CNN-based and PSSM profiles to perform robust SNARE detection on high-throughput and imbalanced data.

### Replication of Study

The main purpose of this study is to single out the SNARE proteins. However, this framework may be applied to discover different kinds of proteins in the field of bioinformatics. To spread our work and contribute to future studies, we made our work publicly available at https://github.com/khanhlee/snare-mcnn. We look forward to exchanging ideas and discussing with other researchers and developers to advance our work in the future.

## Conclusions

SNARE proteins play a key role in the biological immune system to resist microbial infection. Thus, it is necessary to develop models that can assist the detection of these proteins. With this study, we addressed the shortcomings of previously proposed strategies, i.e., (1) traditional machine learning algorithms could not retrieve the hidden information from input protein sequence, and (2) conventional CNN could not retain the sequencing order provided by PSSM profiles. In this study, we proposed a novel framework based on PSSM profiles and multiscan CNN to recognize the SNARE sequences among other general proteins. Fivefold cross-validation was performed on the training set with different feature extractors involved. We also conducted many experiments to compare multiscan CNN with other traditional machine learning classifiers. After generating the optimal model with multiscan CNN and PSSM profiles, we validated its performance on an independent dataset. The experimental measurements yielded by our framework surpassed the existing machine learning methods and advanced the previous CNN strategy. To our knowledge, this is the first report of using multiscan CNN and PSSM profiles to accomplish these tasks.

Altogether, we have demonstrated the competence of our novel framework in identifying the SNARE proteins. Furthermore, our approach may facilitate discovering new functions of other proteins. Future research may include combining more feature extraction methods or unearthing new proteins with hidden or undiscovered functions.

## Notes

Our source codes and datasets are freely available at https://github.com/khanhlee/snare-mcnn..

## References

[ref1] Jahn R.; Scheller R. H. SNAREs–engines for membrane fusion. Nat. Rev. Mol. Cell Biol. 2006, 7, 631–643. 10.1038/nrm2002. 16912714

[ref2] Wickner W.; Schekman R. Membrane fusion. Nat. Struct. Mol. Biol. 2008, 15, 658–664. 10.1038/nsmb.1451. 18618939 PMC2488960

[ref3] Söllner T.; Bennett M. K.; Whiteheart S. W.; Scheller R. H.; Rothman J. E. A protein assembly-disassembly pathway in vitro that may correspond to sequential steps of synaptic vesicle docking, activation, and fusion. Cell 1993, 75, 409–418. 10.1016/0092-8674(93)90376-2. 8221884

[ref4] Weber T.; Zemelman B. V.; McNew J. A.; Westermann B.; Gmachl M.; Parlati F.; Söllner T. H.; Rothman J. E. SNAREpins: minimal machinery for membrane fusion. Cell 1998, 92, 759–772. 10.1016/S0092-8674(00)81404-X. 9529252

[ref5] Trimble W. S.; Cowan D. M.; Scheller R. H. VAMP-1: a synaptic vesicle-associated integral membrane protein. Proc. Natl. Acad. Sci. U.S.A. 1988, 85, 4538–4542. 10.1073/pnas.85.12.4538. 3380805 PMC280466

[ref6] Oyler G. A.; Higgins G. A.; Hart R. A.; Battenberg E.; Billingsley M.; Bloom F. E.; Wilson M. C. The identification of a novel synaptosomal-associated protein, SNAP-25, differentially expressed by neuronal subpopulations. J. Cell Biol. 1989, 109, 3039–3052. 10.1083/jcb.109.6.3039. 2592413 PMC2115928

[ref7] Inoue A.; Obata K.; Akagawa K. Cloning and sequence analysis of cDNA for a neuronal cell membrane antigen, HPC-1. J. Biol. Chem. 1992, 267, 10613–10619. 10.1016/S0021-9258(19)50061-8. 1587842

[ref8] Bennett M. K.; Calakos N.; Scheller R. H. Syntaxin: a synaptic protein implicated in docking of synaptic vesicles at presynaptic active zones. Science 1992, 257, 255–259. 10.1126/science.1321498. 1321498

[ref9] Kutay U.; Hartmann E.; Rapoport T. A. A class of membrane proteins with a C-terminal anchor. Trends Cell Biol. 1993, 3, 72–75. 10.1016/0962-8924(93)90066-A. 14731773

[ref10] Hess D. T.; Slater T. M.; Wilson M. C.; Skene J. The 25 kDa synaptosomal-associated protein SNAP-25 is the major methionine-rich polypeptide in rapid axonal transport and a major substrate for palmitoylation in adult CNS. J. Neurosci. 1992, 12, 4634–4641. 10.1523/JNEUROSCI.12-12-04634.1992. 1281490 PMC6575770

[ref11] Ulloa F.; Gonzalez-Junca A.; Meffre D.; Barrecheguren P. J.; Martinez-Marmol R.; Pazos I.; Olive N.; Cotrufo T.; Seoane J.; Soriano E. Blockade of the SNARE protein syntaxin 1 inhibits glioblastoma tumor growth. PLoS One 2015, 10, e0119707 10.1371/journal.pone.0119707. 25803850 PMC4372377

[ref12] Meng J.; Wang J. Role of SNARE proteins in tumourigenesis and their potential as targets for novel anti-cancer therapeutics. Biochim. Biophys. Acta, Rev. Cancer 2015, 1856, 1–12. 10.1016/j.bbcan.2015.04.002. 25956199

[ref13] Che Y.; Siprashvili Z.; Kovalski J. R.; Jiang T.; Wozniak G.; Elcavage L.; Khavari P. A. KRAS regulation by small non-coding RNAs and SNARE proteins. Nat. Commun. 2019, 10, 5118 10.1038/s41467-019-13106-4. 31712554 PMC6848142

[ref14] Fader C. M.; Sánchez D. G.; Mestre M. B.; Colombo M. I. TI-VAMP/VAMP7 and VAMP3/cellubrevin: two v-SNARE proteins involved in specific steps of the autophagy/multivesicular body pathways. Biochim. Biophys. Acta, Mol. Cell Res. 2009, 1793, 1901–1916. 10.1016/j.bbamcr.2009.09.011. 19781582

[ref15] Burgoyne R. D.; Morgan A. Chaperoning the SNAREs: a role in preventing neurodegeneration?. Nat. Cell Biol. 2011, 13, 8–9. 10.1038/ncb0111-8. 21173802

[ref16] Johnson R. D.; Oliver P. L.; Davies K. E. SNARE proteins and schizophrenia: linking synaptic and neurodevelopmental hypotheses. Acta Biochim. Pol. 2008, 55, 619–628. 10.18388/abp.2008_3022. 18985177

[ref17] Chen F.; Chen H.; Chen Y.; Wei W.; Sun Y.; Zhang L.; Cui L.; Wang Y. Dysfunction of the SNARE complex in neurological and psychiatric disorders. Pharmacol. Res. 2021, 165, 105469 10.1016/j.phrs.2021.105469. 33524541

[ref18] Gao J.; Kurre R.; Rose J.; Walter S.; Fröhlich F.; Piehler J.; Reggiori F.; Ungermann C. Function of the SNARE Ykt6 on autophagosomes requires the Dsl1 complex and the Atg1 kinase complex. EMBO Rep. 2020, 21, e50733 10.15252/embr.202050733. 33025734 PMC7726795

[ref19] Wu S.-R. J.; Khoriaty R.; Kim S. H.; O’Shea K. S.; Zhu G.; Hoenerhoff M.; Zajac C.; Oravecz-Wilson K.; Toubai T.; Sun Y. SNARE protein SEC. 22B regulates early embryonic development. Sci. Rep. 2019, 9, 11434 10.1038/s41598-018-37186-2. 31391476 PMC6685974

[ref20] Lu B. The destructive effect of botulinum neurotoxins on the SNARE protein: SNAP-25 and synaptic membrane fusion. PeerJ 2015, 3, e1065 10.7717/peerj.1065. 26157630 PMC4493708

[ref21] Chen W.; Lv H.; Nie F.; Lin H. i6mA-Pred: identifying DNA N6-methyladenine sites in the rice genome. Bioinformatics 2019, 35, 2796–2800. 10.1093/bioinformatics/btz015. 30624619

[ref22] Le N. Q. K.; Nguyen V.-N. SNARE-CNN: a 2D convolutional neural network architecture to identify SNARE proteins from high-throughput sequencing data. PeerJ Comput. Sci. 2019, 5, e177 10.7717/peerj-cs.177. PMC7924420 33816830

[ref23] Gao X.; Li G. A KNN model based on manhattan distance to identify the SNARE proteins. IEEE Access 2020, 8, 112922–112931. 10.1109/ACCESS.2020.3003086.

[ref24] Li G. Identification of SNARE proteins through a novel hybrid model. IEEE Access 2020, 8, 117877–117887. 10.1109/ACCESS.2020.3004446.

[ref25] Zhang Z.; Gong Y.; Gao B.; Li H.; Gao W.; Zhao Y.; Dong B. SNAREs-SAP: SNARE Proteins Identification With PSSM Profiles. Front Genet. 2021, 12, 809001 10.3389/fgene.2021.809001. 34987554 PMC8721734

[ref26] Fukushima K. A self-organizing neural network model for a mechanism of pattern recognition unaffected by shift in position. Biol. Cybern. 1980, 36, 193–202. 10.1007/BF00344251. 7370364

[ref27] LeCun Y.; Boser B.; Denker J. S.; Henderson D.; Howard R. E.; Hubbard W.; Jackel L. D. Backpropagation applied to handwritten zip code recognition. Neural Comput. 1989, 1, 541–551. 10.1162/neco.1989.1.4.541.

[ref28] Senior A. W.; Evans R.; Jumper J.; Kirkpatrick J.; Sifre L.; Green T.; Qin C.; Žídek A.; Nelson A. W.; Bridgland A.; et al. Improved protein structure prediction using potentials from deep learning. Nature 2020, 577, 706–710. 10.1038/s41586-019-1923-7. 31942072

[ref29] Gessulat S.; Schmidt T.; Zolg D. P.; Samaras P.; Schnatbaum K.; Zerweck J.; Knaute T.; Rechenberger J.; Delanghe B.; Huhmer A.; et al. Prosit: proteome-wide prediction of peptide tandem mass spectra by deep learning. Nat. Methods 2019, 16, 509–518. 10.1038/s41592-019-0426-7. 31133760

[ref30] LeCun Y.; Bengio Y.; Hinton G. Deep learning. Nature 2015, 521, 436–444. 10.1038/nature14539. 26017442

[ref31] Ho Q.-T.; Le N. Q. K.; Ou Y.-Y. *m*CNN-ETC: identifying electron transporters and their functional families by using multiple windows scanning techniques in convolutional neural networks with evolutionary information of protein sequences. Brief Bioinform. 2022, 23, bbab352 10.1093/bib/bbab352. 34472594

[ref32] Seo S.; Oh M.; Park Y.; Kim S. DeepFam: deep learning based alignment-free method for protein family modeling and prediction. Bioinformatics 2018, 34, i254–i262. 10.1093/bioinformatics/bty275. 29949966 PMC6022622

[ref33] Consortium U. UniProt: a hub for protein information. Nucleic Acids Res. 2015, 43, D204–D212. 10.1093/nar/gku989. 25348405 PMC4384041

[ref34] Altschul S. F.; Madden T. L.; Schäffer A. A.; Zhang J.; Zhang Z.; Miller W.; Lipman D. J. Gapped BLAST and PSI-BLAST: a new generation of protein database search programs. Nucleic Acids Res. 1997, 25, 3389–3402. 10.1093/nar/25.17.3389. 9254694 PMC146917

[ref35] Jones D. T. Protein secondary structure prediction based on position-specific scoring matrices. J. Mol. Biol. 1999, 292, 195–202. 10.1006/jmbi.1999.3091. 10493868

[ref36] Jeong J. C.; Lin X.; Chen X.-W. On position-specific scoring matrix for protein function prediction. IEEE/ACM Trans. Comput. Biol. Bioinf. 2011, 8, 308–315. 10.1109/TCBB.2010.93. 20855926

[ref37] Le N. Q. K. Fertility-GRU: Identifying Fertility-Related Proteins by Incorporating Deep-Gated Recurrent Units and Original Position-Specific Scoring Matrix Profiles. J. Proteome Res. 2019, 18, 3503–3511. 10.1021/acs.jproteome.9b00411. 31362508

[ref38] Xie D.; Li A.; Wang M.; Fan Z.; Feng H. LOCSVMPSI: a web server for subcellular localization of eukaryotic proteins using SVM and profile of PSI-BLAST. Nucleic Acids Res. 2005, 33, W105–W110. 10.1093/nar/gki359. 15980436 PMC1160120

[ref39] Chen T.-R.; Juan S.-H.; Huang Y.-W.; Lin Y.-C.; Lo W.-C. A secondary structure-based position-specific scoring matrix applied to the improvement in protein secondary structure prediction. PLoS One 2021, 16, e0255076 10.1371/journal.pone.0255076. 34320027 PMC8318245

[ref40] Pruitt K. D.; Tatusova T.; Maglott D. R. NCBI reference sequences (RefSeq): a curated non-redundant sequence database of genomes, transcripts and proteins. Nucleic Acids Res. 2007, 35, D61–D65. 10.1093/nar/gkl842. 17130148 PMC1716718

[ref41] Chou K. C. A novel approach to predicting protein structural classes in a (20–1)-D amino acid composition space. Proteins 1995, 21, 319–344. 10.1002/prot.340210406. 7567954

[ref42] Chen Z.; Zhao P.; Li F.; Leier A.; Marquez-Lago T. T.; Wang Y.; Webb G. I.; Smith A. I.; Daly R. J.; Chou K.-C.; Song J. iFeature: a python package and web server for features extraction and selection from protein and peptide sequences. Bioinformatics 2018, 34, 2499–2502. 10.1093/bioinformatics/bty140. 29528364 PMC6658705

[ref43] Nguyen T.-T.-D.; Ho Q.-T.; Tarn Y.-C.; Ou Y.-Y. MFPS_CNN: Multi-filter Pattern Scanning from Position-specific Scoring Matrix with Convolutional Neural Network for Efficient Prediction of Ion Transporters. Mol. Inf. 2022, 41, 2100271 10.1002/minf.202100271. 35322557

[ref44] Alipanahi B.; Delong A.; Weirauch M. T.; Frey B. J. Predicting the sequence specificities of DNA-and RNA-binding proteins by deep learning. Nat. Biotechnol. 2015, 33, 831–838. 10.1038/nbt.3300. 26213851

[ref45] Chawla N. V.; Bowyer K. W.; Hall L. O.; Kegelmeyer W. P. SMOTE: synthetic minority over-sampling technique. J. Artif. Intell. Res. 2002, 16, 321–357. 10.1613/jair.953.

[ref46] Höglund A.; Dönnes P.; Blum T.; Adolph H.-W.; Kohlbacher O. MultiLoc: prediction of protein subcellular localization using N-terminal targeting sequences, sequence motifs and amino acid composition. Bioinformatics 2006, 22, 1158–1165. 10.1093/bioinformatics/btl002. 16428265

[ref47] Bhasin M.; Raghava G. ESLpred: SVM-based method for subcellular localization of eukaryotic proteins using dipeptide composition and PSI-BLAST. Nucleic Acids Res. 2004, 32, W414–W419. 10.1093/nar/gkh350. 15215421 PMC441488

[ref48] Bhasin M.; Raghava G. P. Classification of nuclear receptors based on amino acid composition and dipeptide composition. J. Biol. Chem. 2004, 279, 23262–23266. 10.1074/jbc.M401932200. 15039428

[ref49] Chicco D.; Jurman G. The advantages of the Matthews correlation coefficient (MCC) over F1 score and accuracy in binary classification evaluation. BMC Genomics 2020, 21, 1–13. 10.1186/s12864-019-6413-7. PMC6941312 31898477

[ref50] Boughorbel S.; Jarray F.; El-Anbari M. Optimal classifier for imbalanced data using Matthews Correlation Coefficient metric. PLoS One 2017, 12, e0177678 10.1371/journal.pone.0177678. 28574989 PMC5456046

[ref51] Van der Maaten L.; Hinton G. Visualizing data using t-SNE. J. Mach. Learn. Res. 2008, 9, 2579–2605.

[ref52] McInnes L.; Healy J.; Saul N.; Großberger L. UMAP: Uniform Manifold Approximation and Projection. J. Open Source Software 2018, 3, 861 10.21105/joss.00861.

[ref53] Lan L.; Djuric N.; Guo Y.; Vucetic S. MS-k NN: protein function prediction by integrating multiple data sources. BMC Bioinf. 2013, 14, S8 10.1186/1471-2105-14-S3-S8. PMC3584913 23514608

[ref54] Cai C.; Han L. Y.; Ji Z. L.; Chen X.; Chen Y. Z. SVM-Prot: web-based support vector machine software for functional classification of a protein from its primary sequence. Nucleic Acids Res. 2003, 31, 3692–3697. 10.1093/nar/gkg600. 12824396 PMC169006

[ref55] Tng S. S.; Le N. Q. K.; Yeh H.-Y.; Chua M. C. H. Improved Prediction Model of Protein Lysine Crotonylation Sites Using Bidirectional Recurrent Neural Networks. J. Proteome Res. 2022, 21, 265–273. 10.1021/acs.jproteome.1c00848. 34812044

[ref56] Mamoshina P.; Vieira A.; Putin E.; Zhavoronkov A. Applications of deep learning in biomedicine. Mol. Pharmaceutics 2016, 13, 1445–1454. 10.1021/acs.molpharmaceut.5b00982. 27007977

[ref57] Le N. Q. K. Potential of deep representative learning features to interpret the sequence information in proteomics. Proteomics 2022, 22, 2100232 10.1002/pmic.202100232. 34730875

